# The P2X7 Receptor Is Shed Into Circulation: Correlation With C-Reactive Protein Levels

**DOI:** 10.3389/fimmu.2019.00793

**Published:** 2019-04-12

**Authors:** Anna Lisa Giuliani, Merhej Berchan, Juana M. Sanz, Angelina Passaro, Stefano Pizzicotti, Valentina Vultaggio-Poma, Alba Clara Sarti, Francesco Di Virgilio

**Affiliations:** ^1^Department of Morphology, Surgery and Experimental Medicine, University of Ferrara, Ferrara, Italy; ^2^Medical Science Department, University of Ferrara, Ferrara, Italy; ^3^Laboratory Division of the S. Anna Hospital, University of Ferrara, Ferrara, Italy

**Keywords:** cytokines, extracellular ATP, inflammation, microvesicles, purinergic signaling

## Abstract

The P2X7 receptor (P2X7R) is a key pro-inflammatory plasma membrane receptor responsible for NLRP3 inflammasome activation and IL-1β release. Various inflammatory plasma membrane receptors (e.g., IL-1 type I receptor, TNF type I and II receptors, IL-2 receptor) are shed under different pathophysiological conditions. In the present study, we show that the full length P2X7R is released into circulation in patients as well as in healthy subjects. Blood levels of shed P2X7R (sP2X7R) correlate to those of the inflammatory marker C reactive protein (CRP). Blood sP2X7R ranged from 16.74 to 82.17 ng/L, mean ± SE 40.97 ± 3.82 (*n* = 26) in healthy subjects, from 33.1 to 484.0 ng/L, mean ± SE 114.78 ± 12.22 (*n* = 45) in patients with CRP <3 mg/L, and from 63.65 to 1092.3 ng/L, mean ± SE 204.2 ± 30.94 (*n* = 42) in patients with CRP >3 mg/L. sP2X7R in plasma was largely associated to microvesicles/microparticles. Peripheral blood monocytes from healthy subjects released sP2X7R upon stimulation with the semi-selective P2X7R agonist benzoyl ATP. These data show that the P2X7R can be released into circulation, and that its blood levels increase in various disease conditions.

## Introduction

Plasma membrane receptors for cytokines or growth factors can be shed due to protease-mediated cleavage, or released in association with plasma membrane-derived microvesicles/microparticles (MVs/MPs) (1, 2), thus deeply affecting signaling by the cognate cytokine (3). Although not routinely used in the diagnosis of inflammation, measurement of soluble cytokine receptor is gaining interest for the differential diagnosis of selected immune-related disorders.

The P2X7R has been implicated in several disease conditions, ranging from infections (4) to neurodegeneration (5), from autoimmune diseases (6) to cancer (7). Increasing attention is being paid to the potential involvement of this receptor in neuropsychiatric disorders (8, 9). A substantial effort has been produced by Pharma Industry to develop potent and selective P2X7R blockers for the treatment of chronic inflammatory diseases, but Phase II/III clinical studies failed to provide clear demonstration of the usefulness of P2X7R blockade (10). Nevertheless, preclinical indications supporting participation of extracellular ATP and the P2X7R in inflammation are compelling (11–13). It is well known that the P2X7 receptor (P2X7R) is a potent stimulant of NLRP3 inflammasome activation and IL-1β release (14, 15), besides being involved in T lymphocyte proliferation and differentiation (16). Gorecki and co-workers showed that P2X7R activation triggers release of matrix metallo-proteinase-2 (MMP-2), which in turn cleaves and releases a truncated form of P2X7R itself (17). However, it is not known whether the P2X7R is released into circulation. In addition, a technically easy and affordable laboratory procedure for the measurement of shed P2X7R (sP2X7R) has not been validated yet.

It has been previously reported that full length P2X7 subunits are associated to MVs/MPs released by immune cells (18), or that a C-terminal cleaved form can be shed following MMP-2 activation (17). However, there are no measurement of P2X7R levels in blood. Increased circulating levels of the P2X7R might be associated to inflammation, and possibly be a useful diagnostic or prognostic marker. In the present study, we have used a recently developed commercial ELISA kit to detect sP2XR7 in the circulation of healthy controls and of subjects admitted to the S. Anna Hospital, University of Ferrara, with various diagnosis. Blood sP2X7R levels were correlated to C reactive protein (CRP) concentration. Based on previous evidence on the pathophysiological role of the P2X7R (10, 19), patients affected by infections, cancer, ischemia and traumatic and autoimmune diseases were included. Our data also show that the full-length P2X7R was released into circulation in association to plasma membrane-derived MVs/MPs. sP2X7R blood levels were comprised within the 16.74 to 82.17 ng/L, and 33.10 to 1092.30 ng/L range in healthy and diseased subjects, respectively. Determination of sP2X7R blood levels might provide a novel diagnostic procedure to monitor inflammation.

## Materials and Methods

### Sample Collection

#### Control Blood Samples

Blood samples were obtained from 30 anonymous healthy controls (age range 25–65 years) from the Ferrara blood donor bank. Twenty-six samples were directly collected into EDTA-coated tubes. Plasma was obtained by centrifugation at 4°C for 15 min at 1000xg, and stored at −80C° until use. Freezing/thawing cycles were carefully avoided. Four samples were split into EDTA-coated or EDTA-free tubes to compare sP2X7R levels in plasma or serum from the same subjects. Serum was prepared by allowing blood coagulation for 15 min at room temperature, followed by clot removal at 4°C for 15 min at 1500xg. Each plasma or serum sample was then split into two aliquots, which were stored overnight either at 4° or at −80°C, to check the effect of storage temperature on sP2X7R measurement. The protocol was approved by the Ethical Committee of Ferrara district (Study n. 170891). All subjects gave written informed consent in accordance with the Declaration of Helsinki.

### Serum Samples

Eighty-seven anonymous serum samples (39 females and 48 males, mean age 68 years, range 27–94 years), from patients admitted at the S. Anna Hospital (Ferrara) were stratified according to the diagnosis at admission into four groups: infectious diseases (*n* = 42), cancer (*n* = 16), ischemic heart or brain disease (*n* = 10) and others (trauma and autoimmune diseases) (*n* = 19). The protocol was approved by the Ethical Committee of Ferrara district (Study n. 040408). Sera, kept at 4°C until sP2X7R measurement, were tested within few hours from CRP determination.

### Isolation of Microvesicles/Microparticles (MVs/MPs) From Plasma

Eight freshly prepared plasma samples were used to isolate MVs/MPs by centrifugation for 15 min at 2200xg (4°C) to get rid of platelets, followed by centrifugation for 60 min at 14000xg (4°C). In addition, MVs/MPs were also isolated from four plasma and four serum samples by centrifugation for 90 min at 100000xg (4°C) after the initial centrifugation at 2200xg. MVs/MPs enriched pellets and MVs/MPs-deprived plasma and sera were collected and stored at −80°C until analysis by SDS-PAGE and Western blot, or ELISA.

### Separation of Platelets and Peripheral Blood Mononuclear Cells (PBMCs)

Platelets were isolated by centrifugation of plasma at 2200xg (4°C) for 15 min. Peripheral blood mononuclear cells (PBMCs) were isolated as previously described (20). Non-adherent cells were removed and the adherent mononuclear cells (monocytes/macrophages) were incubated in 10% FBS-supplemented RPMI under the different conditions described in the figure legend.

### Cell Cultures

To validate sP2X7R measurement in serum and plasma, three human cell lines were used: wild type HEK293 cells (wt-HEK293), HEK293 cells stably transfected with the human P2X7R (HEK293-P2X7R), and ACN (human neuroblastoma) cells. wt-HEK293 cells that do not express the endogenous P2X7R were used as negative control, while ACN cells, that over-express the endogenous P2X7R, and HEK293-P2X7R were used as positive controls. wt-HEK293, HEK293-P2X7R and ACN cells were cultured as previously described (21). For ELISA analysis, cell monolayers were scraped off the plate, suspended in PBS at a concentration of 10^8^ cells/ml, and lysed by two freeze-thaw cycles. Cell lysates were centrifuged for 5 min at 5000xg at 4°C and supernatants collected and immediately assayed.

### Measurement of sP2X7R by ELISA

sP2X7R was measured in plasma, sera, cell lysates and supernatants from monocyte cultures with the Human P2X purinoceptor 7 (P2RX7) ELISA kit (Cusabio, Houston, TX, USA) following manufacturer's instructions. Optical density was determined at 450 nm by a Multiskan FC spectrophotometer (Thermo Scientific).

### SDS-PAGE and Western Blot

For SDS-PAGE/Western Blot analysis, cell lysates, MVs/MPs and platelets were solubilized in RIPA-buffer (150 mM NaCl/0.1% SDS/0.5% Na-deoxycholate/1% Triton X-100/50 mM Tris, pH 7.2). Protein concentration was measured with the Bradford assay. Ten μg of protein were loaded onto a 4–12% Bis-Tris Nu-PAGE gel (Invitrogen, Life Technologies, Carlsbad, CA, USA). Proteins were blotted for 3 h onto a nitrocellulose membrane (Amersham, GE Healthcare, Chicago, IL, USA). Non-specific binding sites were blocked with 5% skim milk in TBS buffer (10 mM Tris-HCl, 150 mM NaCl, pH 8.0). Nitrocellulose strips were then incubated overnight at 4°C either with a rabbit anti-human P2X7R antibody (Millipore, Burlington, MA, USA, cat n AB5246) diluted 1:600, or with a rabbit anti-human actin antibody (Sigma-Aldrich), diluted 1:1000 in TBS-t buffer (TBS plus 0.1% Tween 20 and 1% BSA). Secondary antibody was a HRP-conjugated goat anti-rabbit IgG (H+L) antibody (BioRad, Hercules, CA, USA) at a 1:3000 dilution in TBS-t buffer. Finally, ECL reagent (Invitrogen, Life Technologies) was used for detection with a LI-COR blot scanner (LI-COR Biosciences, Lincoln, NE, USA).

### C Reactive Protein (CRP) Measurement

CRP was measured at the Laboratory Division of the S. Anna Hospital with the immune-turbidimetric kit CRP OSR6147 (Beckman Coulter, Brea, CA, USA).

### Statistics

Data were expressed as means ± SE. Variables that did not follow normal distribution were log transformed before statistical analyses. One way ANOVA or Kruskal-Wallis tests were used for comparisons among groups and unpaired *t*-test or Mann-Whitney test, paired *t*-test or Wilcoxon matched-pairs signed rank test were performed for comparisons between groups. Spearman's correlation coefficient was used to test the association between P2X7R, age and CRP. This test was followed by multiple regression analysis in order to check the independence of the observed simple associations. All tests were considered significant for a *p*-value <0.05. Statistical analysis was performed using SPSS 22.0 software (SPSS, Chicago, IL, USA).

## Results

### Validation of the Test: Detection of Shed P2X7R (sP2X7R) in Plasma, Serum and Cell Lysates

**Figure 1:**
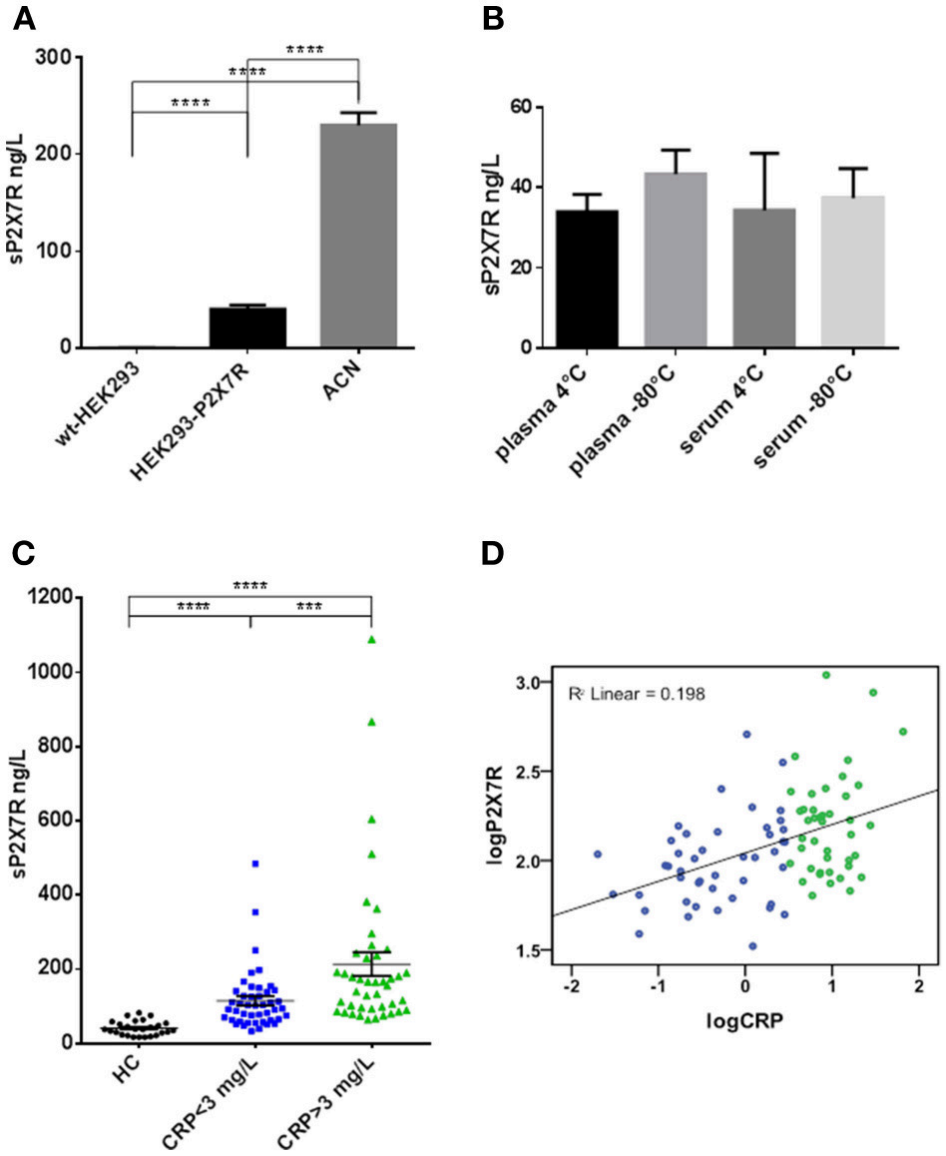
The P2X7R was present in human plasma and serum, its concentration was higher in diseased subjects and correlated with CRP. **(A)** ELISA analysis for sP2X7R of lysates from 10^8^ cells/ml of wt-HEK293, HEK293-P2X7R, and ACN neuroblastoma cells. Data are means ± SE of results from 3 separate experiments. Differences are statistically significant (One-way ANOVA, *p* < 0.0001; unpaired t-test between groups: ****p < 0.0001). **(B)** Effect of storage conditions on sP2X7R detection by ELISA in plasma and serum samples from 4 healthy subjects. Differences are not statistically significant. **(C)** sP2X7R levels were measured by ELISA in serum samples from 26 healthy control subjects (HC), 45 patients with CRP <3 mg/L and 42 patients with CRP >3 mg/L. Data are means ± SE. Mann Whitney test between groups: ***p < 0.001; ****p < 0.0001. **(D)** Linear regression analysis between log-transformed CRP and sP2X7R concentrations in serum samples from 87 diseased subjects. Color coding in **(C,D)**: blue, CRP <3 mg/L, green. CRP >3 mg/L.

Plasma, serum and cell lysate samples were tested for the presence of sP2X7R by ELISA. sP2X7R concentration in plasma from healthy subjects (*n* = 26) ranged from 16.7 to 82.17 ng/L (mean ± SE 40.97 ± 3.82). Due to high protein content of plasma, sP2X7R detection by Western blot produced unreliable results since most proteins run in the 60 to 80 kDa range, thus overshadowing the P2X7 band. Therefore, specificity of the ELISA assay was validated with cell lysates from wt-HEK293 (P2X7R-null), HEK293-P2X7R, and ACN cells. The assay showed virtually no P2X7R immunoreactivity in wt-HEK293 cells (<0.2 ng/L), and a robust signal in HEK293-P2X7R and ACN cells, 38 and 220 ng/L, respectively (Figure 1A). No significant difference was observed in sP2X7R levels in plasma or serum samples prepared from the same individuals, whether stored at 4° or −80° C (Figure 1B).

### Blood sP2X7R Levels and Correlation With CRP

In healthy control subjects (HC), sP2X7R was comprised in the 16.7 to 82.17 ng/L range (mean ± SE = 40.97 ± 3.82, Figure 1C). We then investigated the correlation with CRP. Eighty-seven anonymous serum samples (39 females and 48 males, mean age 68 years, range 27–94 years) were randomly and anonymously received from the Laboratory Division of the S. Anna Hospital. In these samples, sP2X7R levels ranged from 33.10 to 1092.30 (mean ± SE = 157.9 ± 16.82), and correlated with age (Spearman correlation Coefficient 0.212, *p* = 0.048) and CRP concentration (Spearman correlation Coefficient 0.412, *p* = 0.000). To further assess the strength of sP2X7R/CRP correlation, we performed a linear regression analysis (Figure 1D) (R^2^ linear = 0.198) as well as a multivariate regression analysis, adjusting for sex, age, and diagnosis sub-group (β-coefficient =0.431, *p* = 0.000). After this adjustment, R^2^ linear slightly increased from 0.198 to 0.213.

Samples were split into 2 groups on the basis of CRP values: 45 samples with CRP <3 mg/L, and 42 samples with CRP>3 mg/L. The 3 mg/L cut-off value was chosen as this is the CRP threshold suggestive of systemic inflammation (22). Serum sP2X7R levels in the population identified by CRP > 3 mg/L were about twice higher than those in the <3 mg/L CRP group, mean ± SE = 204.2 ± 30.94 (n = 42), vs. 114.8 ± 12.22, (*n* = 45), respectively, and significantly different (Mann Whitney test between groups: *p* < 0.001) (Figure 1C).

**Figure 2:**
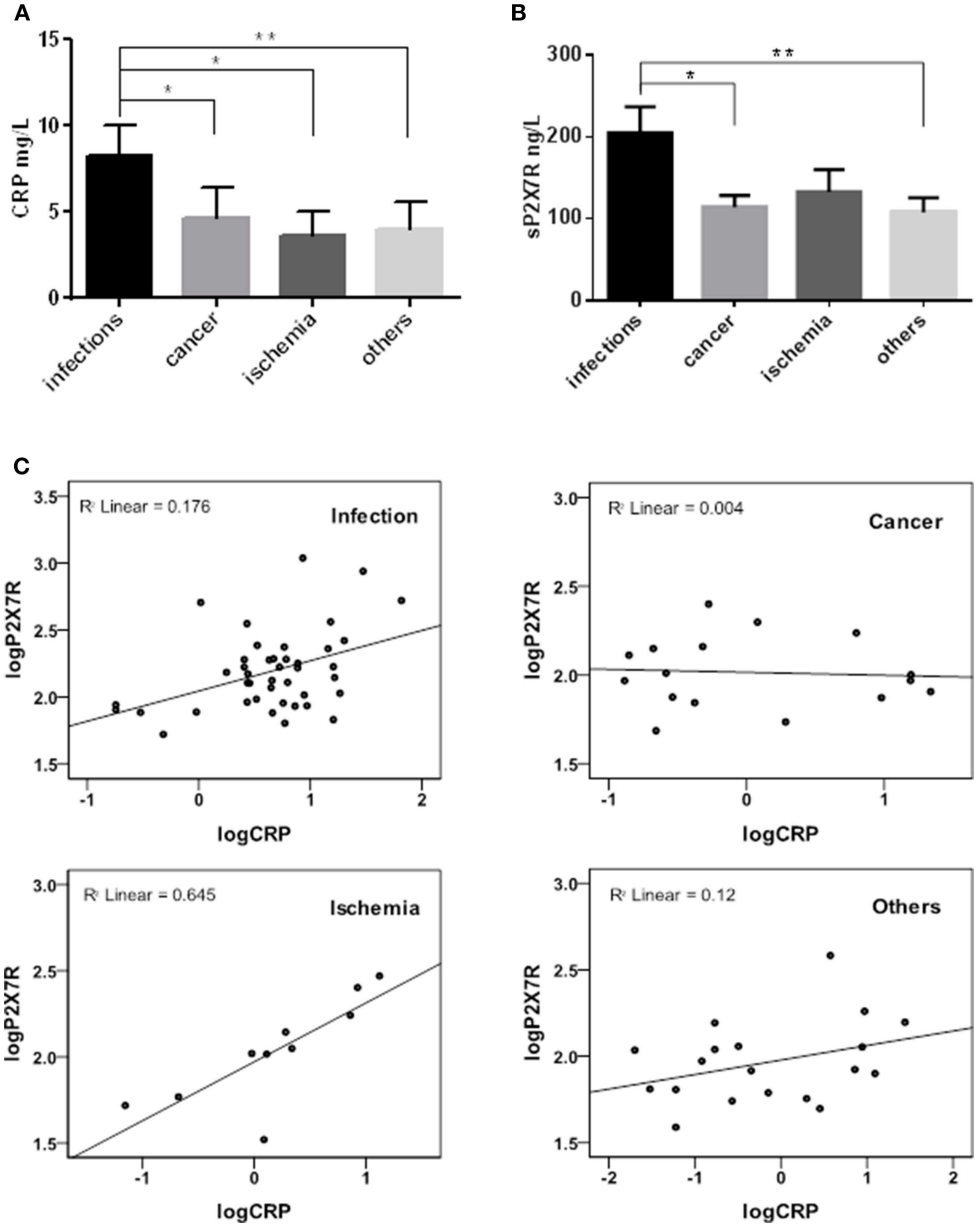
sP2X7R was differentially concentrated in different diagnosis sub-groups. CRP and sP2X7R were measured in serum samples stratified into four sub-groups according to diagnosis at admission to the hospital: infectious diseases (*n* = 42), cancer (*n* = 16), ischemia (*n* = 10) and others (trauma, autoimmune diseases) (*n* = 19). Data are means ± SE. Only significant differences are shown in the graphs. **(A)** Kruskal-Wallis test *p* = 0.0057; Mann Whitney test between groups: *p < 0.05; **p < 0.01. **(B)** Kruskal-Wallis test *p* = 0.0205; Mann Whitney test between groups: *p < 0.05; **p < 0.01. **(C)** Linear regression analysis between log-transformed CRP and sP2X7R in the different diagnosis sub-groups: ischemia, R^2^ linear = 0.645; infectious diseases, R^2^ linear = 0.176; cancer, R^2^ linear = 0.004; others, R^2^ linear = 0.12.

Samples were then stratified into 4 sub-groups according to diagnosis at admission to hospital: infectious diseases (*n* = 42), cancer (*n* = 16), heart or brain ischemia (*n* = 10), and others (trauma, autoimmune diseases) (*n* = 19), and CRP-sP2X7R correlation in the 4 diagnosis sub-groups analyzed (Figure 2). sP2X7R levels in each of the 4 sub-groups were significantly higher than in healthy controls (Mann Whitney test: *p* < 0.0001). Both CRP and sP2X7R levels were significantly higher in the infectious disease patients vs. the other three subgroups (Figures 2A,B), with the exception of sP2X7R in the ischemia patients (Mann Whitney test: *p* = 0.268). Linear regression analysis showed a strong correlation between log-transformed CRP and sP2X7R in the ischemia subgroup (R^2^ linear = 0.645), and a weaker correlation in the infectious disease subgroup (R^2^ linear = 0.176), while there was basically no correlation in the cancer (R^2^ = 0.004) and “others” (R^2^ = 0.12) sub-groups (Figure 2C).

### Sources of sP2X7R

**Figure 3:**
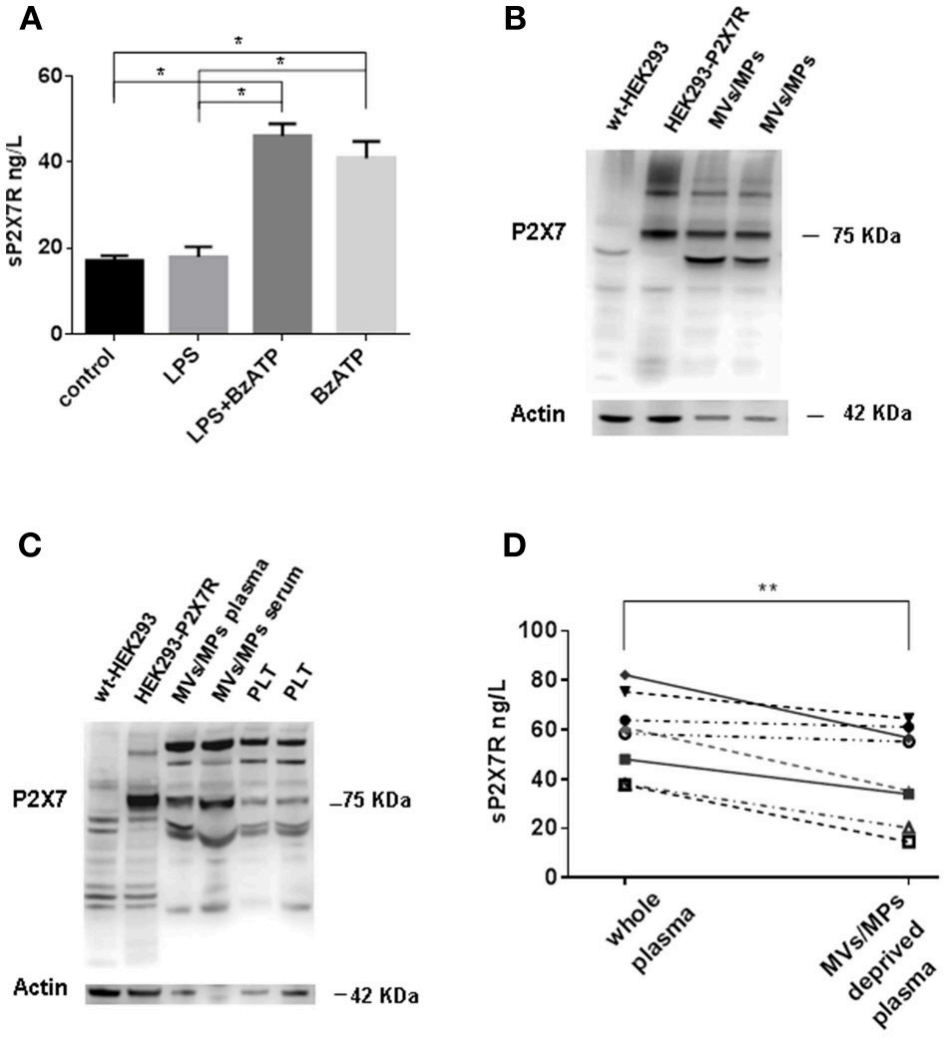
sP2X7R is shed in association with MVs/MPs by P2X7R stimulation. **(A)** Monocyte-derived macrophages were isolated from blood samples of 4 healthy control subjects as described in Methods and incubated in 10% FBS-supplemented RPMI under the following conditions: (1) no additions (control) for 5 h; (2) 1 μg/ml LPS for 4 h; (3) 1 μg/ml LPS for 4 h, followed by 300 μM BzATP for 1 h; (4) no additions for 4 h, followed by 300 μM BzATP for 1 h. Data are means ± SE of data from 4 separate experiments. Only significant differences are shown in the graph. Kruskal-Wallis test *p* = 0.0008. Mann Whitney test between groups: *p < 0.05. **(B)** MVs/MPs were isolated from two plasma samples by centrifugation at 2200xg followed by 14000xg. Ten μg of protein were loaded on the SDS-PAGE gel. **(C)** MVs/MPs were isolated from one plasma sample and from one serum sample by centrifugation at 2200xg followed by 100000xg ultracentrifugation. Platelets (PLT) were isolated from plasma by centrifugation at 2200xg. See Material and Methods for additional details. Ten μg of proteins were loaded on the SDS-PAGE gel. **(D)** ELISA analysis of sP2X7R content of plasma samples from 8 subjects before and after depletion of MVs/MPs by ultracentrifugation at 14000× g. Wilcoxon matched paired *t*-test: *p* = 0.0078.

A substantial level of sP2X7R was released from blood derived monocytes isolated from healthy subjects under basal conditions (Figure 3A). Challenge with LPS (1 μg/ml) did not further increase sP2X7R release. On the contrary, BzATP (300 μM), whether alone or added to LPS-primed cells, triggered a large sP2X7R release. Stimulation of plasma membrane P2X7R by ATP or BzATP is a potent trigger for shedding of MVs/MPs carrying the P2X7R itself (18). To verify the association of sP2X7R with extracellular micro-particulated material, MVs/MPs were isolated according to established protocols from plasma samples of 8 healthy controls, two of which are shown in Figure 3B. Western blot analysis suggests that the sP2X7R was associated to MVs/MPs. MVs/MPs are a heterogeneous population of particles of different sizes and cellular origin, which might also contain platelets or platelet fragments. Therefore, we performed Western blot analysis of MVs/MPs isolated from plasma or serum by an alternative protocol (final centrifugation at 100,000xg). P2X7R immunoreactivity was tested in parallel in purified platelet preparations. As shown in Figure 3C, the P2X7 band was clearly visible in the MVs/MPs fraction, whether from plasma or serum, while only a faint band was visible in platelet fractions. In the MVs/MPs fraction we detected a lower molecular mass band (about 50 kDa) which was also present in HEK293-P2X7 cells, but it is not clear whether this might be a lower molecular mass variant. Two major higher molecular mass bands are also visible in the MVs/MPs preparation and in the platelet lysates, that might however be non-specific. Plasma analysis by ELISA after MVs/MPs deprivation showed a significant reduction of sP2X7R levels (58.03±5.75 vs. 42.69 ± 6.82, in whole vs. MVs/MPs-deprived plasma, respectively, Wilcoxon matched pairs test *p* = 0.0078) (Figure 3D).

## Discussion

Extracellular nucleotides, first of all ATP, have relevant roles in a great number of physiological and pathological phenomena (23). The P2X7R is an extracellular ATP-activated key inflammatory receptor expressed at high levels by immune and tumor cells (10, 19). In the immune system, the P2X7R has been shown to drive NLRP3 inflammasome activation, T lymphocyte proliferation and differentiation, generation of reactive oxygen species, release of cell-derived microvesicles/microparticles (MVs/MPs) and precipitation of pyroptotic cell death (10). The P2X7R is a homotrimer made by the assembly of identical P2X7 subunits. P2X7 subunits are integral membrane proteins characterized by a large ecto-domain, and N- and C-terminal residues both on the cytoplasmic side of the plasma membrane. P2X7 subunits could in principle be shed by proteolytic cleavage, as shown by Gorecki and co-workers (17). However, P2X7 subunits might also be shed in association with plasma membrane-derived MVs/MPs. Sometime ago, we showed that human dendritic cells stimulated *in vitro* via the P2X7R, release plasma membrane-derived MVs/MPs loaded with various plasma membrane molecules, among which P2X7 subunits (18). Based on immunoreactivity with an antibody raised against the C-terminal tail of the P2X7 subunit, we concluded that this subunit was released as the full-length variant. In the present study, we show that also MVs/MPs isolated from peripheral blood carry the P2X7 subunit as the full-length variant. We don't know if in association with MVs/MPs the P2X7R is present as the trimeric receptor (i.e., P2X7R) or as individual subunits (i.e., P2X7), although previous data showing that dendritic cell-released MVs/MPs are lysed by added ATP (18) suggest that it might be present as P2X7R. Since Western Blots are obviously impractical for routine identification of plasma/serum components, we verified whether a recently made available commercial ELISA kit was suitable for measurement of P2X7R as a shed (sP2X7R) molecule. Our data support the validity of ELISA measurement of sP2X7R in blood as concentrations in plasma and serum were almost perfectly superimposable, and did not differ whether measured in samples kept at 4°C or frozen at −80°C. P2X7R concentration was comprised in the 16.7 to 82.17 ng/L and 33.1 to 1090.00 ng/L range in healthy and diseased subjects, respectively. The ELISA kit also provided an accurate measurement of sP2X7R in cell lysates. Although limited information was available on the epitope specificity of the antibodies in the P2X7-ELISA kit, ability to recognize MVs/MPs-associated P2X7R suggest that they should be directed against epitopes exposed on the outer domain of the receptor, as per manufacturer indication.

The full length molecule was detected in blood, but we cannot exclude that some P2X7R was shed following proteolytic cleavage (17). The extracellular ATP concentration can reach the hundred micromolar level at inflammatory sites (24), i.e., sufficient to activate even the low affinity P2X7R and trigger sP2X7R release. sP2X7R levels were significantly lower in healthy vs. diseased subjects, especially in the patient cohort with CRP >3 mg/L. In this cohort, only 6 out of 42 subjects showed sP2X7R serum levels overlapping with the range of healthy subjects. In the cohort with CRP <3 mg/L, 19 out of 45 patients showed P2X7R levels within the healthy subject concentration range. All diagnosis cohorts screened in this study showed P2X7R serum levels significantly higher (*p* < 0.0001) than healthy subjects, suggesting that P2X7R might be a non-specific disease marker. We found that correlation of sP2X7R blood levels with CRP was widely different depending on the disease. Best correlation was found in patients suffering of ischemia (R^2^ linear = 0.645), while the lowest correlation was found in patients with cancer (R^2^ linear = 0.004). Infectious patients showed an intermediate correlation (R^2^ linear = 0.176). Since all these diseases are accompanied by local or systemic inflammation, and therefore in principle by CRP release and P2X7R activation, these data suggest that depending on the etiology, different inflammatory conditions may promote a differential level of both CRP and sP2X7R release, and ischemia is the disease condition where release of both biomarkers is matched. P2X7R measurement might complement that of CRP in the differential diagnosis of inflammatory conditions of different etiology (i.e., infectious vs. non-infectious). Most circulating cells express the P2X7R and release MVs/MPs, thus it is likely that there are multiple sources of sP2X7R in blood and vessels, endothelial cells included.

Although not routinely used in laboratory practice, blood IL-1β levels are of potential usefulness in the diagnosis of inflammatory diseases (25). sP2X7R determinations might complement and strengthen the diagnostic significance of IL-1β measurements.

At this early stage, the pathophysiological significance of P2X7R shedding is unknown. It might be a mere epiphenomenon of inflammation, but we cannot exclude that fusion of MVs/MPs might transfer the P2X7R to other immune cells at sites distal to the original inflammatory site.

In conclusion, we have shown that the P2X7R is released into the blood in association to MVs/MPs, and that its levels are significantly higher in diseased subjects.

## Ethics Statement

The protocols were approved by the Ethical Committee of Ferrara district (Studies n. 170891 and 040408). All subjects gave written informed consent in accordance with the Declaration of Helsinki.

## Author Contributions

FDV projected this work, planned experiments and wrote the manuscript. ALG was responsible for most of the experiments and for drafting and editing the manuscript. MB collected samples, stratified patients' samples according to diagnosis and performed some of the experiments. JMS was responsible for statistical analysis. AP helped in projecting this work and in the statistical analysis. SP collected samples and stratified patients' samples according to diagnosis. VV-P and ACS performed some of the experiments.

### Conflict of Interest Statement

FDV is a member of the Scientific Advisory Board of Biosceptre Ltd., a UK-based biotech Company involved in the development of P2X7R-targeted therapeutics. The remaining authors declare that the research was conducted in the absence of any commercial or financial relationships that could be construed as a potential conflict of interest.

## References

[1] Coleman LG Jr Maile R Jones SW Cairns BA Crews FT. HMGB1/IL-1beta complexes in plasma microvesicles modulate immune responses to burn injury. PLoS ONE. (2018) 13:e0195335. 10.1371/journal.pone.0195335 29601597 PMC5877880

[2] Zhang S Dai H Zhu L Lin F Hu Z Jing R. Microvesicles packaging IL-1beta and TNF-alpha enhance lung inflammatory response to mechanical ventilation in part by induction of cofilin signaling. Int Immunopharmacol. (2018) 63:74–83. 10.1016/j.intimp.2018.07.034 30075431

[3] Sha Y Markovic-Plese S. Activated IL-1RI signaling pathway induces Th17 cell differentiation via interferon regulatory factor 4 signaling in patients with relapsing-remitting multiple sclerosis. Front Immunol. (2016) 7:543. 10.3389/fimmu.2016.00543 27965670 PMC5126112

[4] Moreira-Souza ACA Almeida-da-Silva CLC Rangel TP Rocha GDC Bellio M Zamboni DS. The P2X7 receptor mediates toxoplasma gondii control in macrophages through canonical NLRP3 inflammasome activation and reactive oxygen species production. Front Immunol. (2017) 8:1257. 10.3389/fimmu.2017.01257 29075257 PMC5643413

[5] Jimenez-Pacheco A Diaz-Hernandez M Arribas-Blazquez M Sanz-Rodriguez A Olivos-Ore LA Artalejo AR. Transient P2X7 receptor antagonism produces lasting reductions in spontaneous seizures and gliosis in experimental temporal lobe epilepsy. J Neurosci. (2016) 36:5920–32. 10.1523/JNEUROSCI.4009-15.2016 27251615 PMC6601816

[6] Faliti CE Gualtierotti R Rottoli E Gerosa M Perruzza L Romagnani A. P2X7 receptor restrains pathogenic Tfh cell generation in systemic lupus erythematosus. J Exp Med. (2019) 216:317–36. 10.1084/jem.20171976 30655308 PMC6363434

[7] De Marchi E Orioli E Pegoraro A Sangaletti S Portararo P Curti A. The P2X7 receptor modulates immune cells infiltration, ectonucleotidases expression and extracellular ATP levels in the tumor microenvironment. Oncogene. (2019). 10.1038/s41388-019-0684-y. [Epub ahead of print]. 30655604 PMC6756114

[8] Bhattacharya A. Recent advances in CNS P2X7 physiology and pharmacology: focus on neuropsychiatric disorders. Front Pharmacol. (2018) 9:30. 10.3389/fphar.2018.00030 29449810 PMC5799703

[9] Horvath G Otrokocsi L Beko K Baranyi M Kittel A Antonio Fritz-Ruenes P. P2X7 receptors drive poly(I:C) induced autism-like behavior in mice. J Neurosci. (2019) 39:2542–61. 10.1523/JNEUROSCI.1895-18.2019 30683682 PMC6435822

[10] Di Virgilio F Dal Ben D Sarti AC Giuliani AL Falzoni S. The P2X7 receptor in infection and inflammation. Immunity. (2017) 47:15–31. 10.1016/j.immuni.2017.06.020 28723547

[11] Adinolfi E Giuliani AL De Marchi E Pegoraro A Orioli E Di Virgilio F. The P2X7 receptor: a main player in inflammation. Biochem Pharmacol. (2017) 151:234–44. 10.1016/j.bcp.2017.12.021 29288626

[12] Di Virgilio F Sarti AC Grassi F. Modulation of innate and adaptive immunity by P2X ion channels. Curr Opin Immunol. (2018) 52:51–9. 10.1016/j.coi.2018.03.026 29631184

[13] Burnstock G Knight GE. The potential of P2X7 receptors as a therapeutic target, including inflammation and tumour progression. Purinergic Signal. (2018) 14:1–18. 10.1007/s11302-017-9593-0 29164451 PMC5842154

[14] Orioli E De Marchi E Giuliani AL Adinolfi E. P2X7 receptor orchestrates multiple signalling pathways triggering inflammation, autophagy and metabolic/trophic responses. Curr Med Chem. (2017) 24:2261–75. 10.2174/0929867324666170303161659 28266268

[15] Giuliani AL Sarti AC Falzoni S Di Virgilio F. The P2X7 receptor-interleukin-1 liaison. Front Pharmacol. (2017) 8:123. 10.3389/fphar.2017.00123 28360855 PMC5353276

[16] Salles EM Menezes MN Siqueira R Borges da Silva H Amaral EP Castillo-Mendez SI. P2X7 receptor drives Th1 cell differentiation and controls the follicular helper T cell population to protect against Plasmodium chabaudi malaria. PLoS Pathog. (2017) 13:e1006595. 10.1371/journal.ppat.1006595 28859168 PMC5597262

[17] Young CNJ Chira N Rog J Al-Khalidi R Benard M Galas L. Sustained activation of P2X7 induces MMP-2-evoked cleavage and functional purinoceptor inhibition. J Mol Cell Biol. (2018) 10:229–42. 10.1093/jmcb/mjx030 28992079

[18] Pizzirani C Ferrari D Chiozzi P Adinolfi E Sandona D Savaglio E. Stimulation of P2 receptors causes release of IL-1beta-loaded microvesicles from human dendritic cells. Blood. (2007) 109:3856–64. 10.1182/blood-2005-06-031377 17192399

[19] Di Virgilio F Sarti AC Falzoni S De Marchi E Adinolfi E. Extracellular ATP and P2 purinergic signalling in the tumour microenvironment. Nat Rev Cancer. (2018) 18:601–18. 10.1038/s41568-018-0037-0 30006588

[20] Falzoni S Munerati M Ferrari D Spisani S Moretti S Di Virgilio F. The purinergic P2Z receptor of human macrophage cells. Characterization and possible physiological role. J Clin Invest. (1995) 95:1207–16. 10.1172/JCI117770 7883969 PMC441459

[21] Adinolfi E Raffaghello L Giuliani AL Cavazzini L Capece M Chiozzi P. Expression of P2X7 receptor increases in vivo tumor growth. Cancer Res. (2012) 72:2957–69. 10.1158/0008-5472.CAN-11-1947 22505653

[22] Kushner I Antonelli MJ. What should we regard as an “elevated” C-reactive protein level? Ann Intern Med. (2015) 163:326. 10.7326/L15-5126 26280429

[23] Giuliani AL Sarti AC Di Virgilio F. Extracellular nucleotides and nucleosides as signalling molecules. Immunol Lett. (2018) 205:16–24. 10.1016/j.imlet.2018.11.006 30439478

[24] Pellegatti P Raffaghello L Bianchi G Piccardi F Pistoia V Di Virgilio F. Increased level of extracellular ATP at tumor sites: in vivo imaging with plasma membrane luciferase. PLoS ONE. (2008) 3:e2599. 10.1371/journal.pone.0002599 18612415 PMC2440522

[25] Slaats J Ten Oever J van de Veerdonk FL Netea MG. IL-1beta/IL-6/CRP and IL-18/ferritin: distinct inflammatory programs in infections. PLoS Pathog. (2016) 12:e1005973. 10.1371/journal.ppat.1005973 27977798 PMC5158075

